# Therapeutic Effects of *Cortex acanthopanacis* Aqueous Extract on Bone Metabolism of Ovariectomized Rats

**DOI:** 10.1155/2012/492627

**Published:** 2012-09-11

**Authors:** Zhiguo Zhang, Jiazi Dong, Meijie Liu, Yan Li, Jinghua Pan, Hong Liu, Wenlai Wang, Dong Bai, Lihua Xiang, Gary G. Xiao, Dahong Ju

**Affiliations:** ^1^Institute of Basic Theory, China Academy of Chinese Medical Sciences, No. 16 Nanxiao Road, Dongzhimennei, Beijing 100700, China; ^2^Functional Genomics & Proteomics Laboratory, Osteoporosis Research Center, Creighton University Medical Center, 601N 30th ST, Suite 6730, Omaha, NE 68131, USA

## Abstract

The aim of this study was to evaluate effects of aqueous extract from *Cortex acanthopanacis* (CAE) on osteoporosis rats induced by ovariectomy (OVX) using aqueous extract from *Folium Epimedii* (FEE) as positive control agent. Three-month-old female rats that underwent OVX were treated with CAE. After 12 weeks, bone mineral density (BMD) and indices of bone histomorphometry of tibia were measured. Levels of protein and mRNA expression of osteoprotegerin (OPG) and receptor activator of nuclear factor kappa-B ligand (RANKL) in tibia were evaluated. In addition, the serum concentrations of osteocalcin (OC), interleukin-1 beta (IL-1**β**), interleukin-6 (IL-6), calcitonin (CT), and parathyroid hormone (PTH) were determined. Administration of CAE significantly prevented OVX-induced rats from gain of the body weight. Treatment with CAE increased bone mass remarkably and showed a significant inhibitory effect on bone resorption by downregulating significantly the expression of RANKL in tibia of OVX rats. Meanwhile, treatment of CAE significantly reduced serum level of IL-1**β** and increased level of CT in OVX rats. This suggests that CAE has the potential to be used as an alternative therapeutic agent for postmenopausal osteoporosis.

## 1. Introduction

Osteoporosis, the most common bone remodeling disease, is defined by a low bone mass and a high risk of fractures. It mainly affects postmenopausal women and elderly men. Osteoporosis is caused by an abnormal bone remodeling, that is, excess of resorption and less formation, resulting in an increased risk of hip and vertebral fracture [1]. The development of bone fragility in postmenopausal women resulted from changes in bone remodeling leading to alteration of trabecular bone volume and architecture [2]. In rats, ovariectomy- (OVX-) induced bone loss can be treated by estradiol. Because of similarities in skeletal responses to estrogen deficiency between rats and humans, the mature OVX rats are considered as a convenient animal model for studying early postmenopause-induced bone loss [3].

Estrogen replacement therapy (ERT) has been an established regime for prevention of postmenopausal bone loss [4], but evidence indicates that long-term unopposed ERT may cause an increased risk of ovarian and endometrial cancer [5]. Thus, alternative therapeutic strategy with a proven efficacy and safety should be developed for the prevention and treatment of osteoporosis.

Chinese herbal medicine has been widely used in clinical practice to treat bone diseases for years and will undoubtedly continue to be used as a cost-effective alternative medicine in China [6].

*Cortex acanthopanacis* (CA) and *Folium Epimedii* (FE) have been thought to have similar action documented by the Chinese ancient literatures and used to strengthen bone for long time in China. Many studies had confirmed the antiosteoporotic effect of FE [7–10], while potential therapeutic effect of CA on osteoporosis induced by estrogen deficiency is unknown yet. The objective of this study was to evaluate the effect of aqueous extract from CA (CAE) on ovariectomized rats, using aqueous extract from FE (FEE) as positive control aqueous extract.

## 2. Materials and Methods

### 2.1. Preparation of Aqueous Extracts

*Cortex acanthopanacis* (CA) is the dried root barks of *Acanthopanax gracilistylus* W. W. Smith produced in China. CA was purchased from Xinxiang Medicinal Herbs Co. Ltd. (Henan, China) and identified and authenticated by an expert herbalist at the Institute of Chinese Materia Medica, China Academy of Chinese Medical Sciences (CACMS).

*Folium Epimedii* (FE) are the dried leaves of *Epimedium brevicornum* Maxim produced in China. CA was purchased from Tianjian Medicinal Herbs Co. Ltd. (Shaanxi, China) and identified and authenticated by an expert herbalist at the Institute of Chinese Materia Medica, CACMS.

Raw CA or FE (250 g) was boiled twice with 4 L of distilled water for two hours under reflux. The aqueous extracts were collected and filtered. The filtrates were then concentrated under reduced pressure at 50°C and lyophilized into powder. The yields of the extraction from herbs were both about 20% (w/w).

### 2.2. High-Performance Liquid Chromatography (HPLC) Analysis

Antiosteoporotic compounds in CAE are unidentified until now. We used markers for the authentication of CA to analyzed chemical characteristics of CAE by HPLC. Icariin was regarded as one of active antiosteoporotic compounds [11, 12]. We used icariin as a marker to analyzed chemical characteristics of FEE by HPLC.

The standard samples of markers, syringin, protocatechuic acid, and icariin were all ordered from the National Institutes for Food and Drug Control (Beijing, China). 

For HPLC detection of the syringin, protocatechuic acid, and icariin, Agilent SB-C18 HPLC column (4.6 × 250 mm, 5 *μ*m, Zorbax, Agilent, Santa Clara, CA) and DAD detector were used. The separating conditions of syringin were as follows: methanol : water : phosphoric acid (20 : 80 : 0.1, V/V/V), and flow rate, 1.0 ml min^−1^. The chromatograms were recorded at 265 nm by monitoring spectra within a wavelength range of 190 to 600 nm. The separating conditions of protocatechuic acid were as follows: methanol : water : phosphoric acid (13 : 87 : 0.1, V/V/V), and flow rate, 1.0 ml min^−1^. The chromatograms were recorded at 260 nm. The separating conditions of icariin were as follows: acetonitrile : water (30 : 70, V/V), and flow rate, 1.0 ml min^−1^. The chromatograms were recorded at 270 nm.

**Figure 1:**
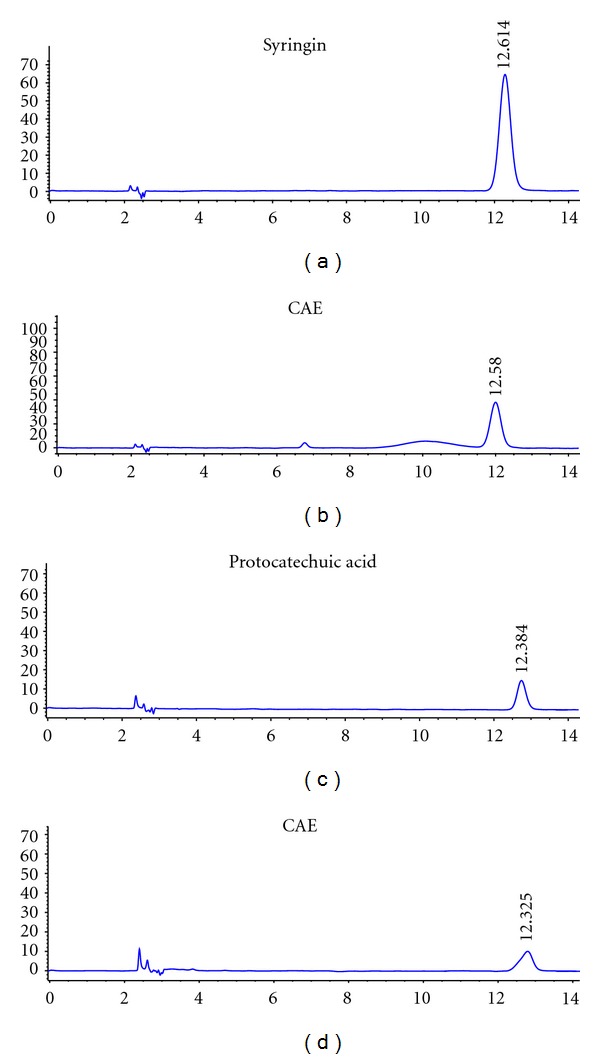
HPLC for the qualitative analysis of syringin and protocatechuic acid in the *Cortex acanthopanacis* extract (CAE). The chromatograms for standard substances and CAE are shown in the upper panel and lower panel, respectively. ((a) and (b)) The chromatograms for standard substance syringin and CAE; ((c) and (d)) the chromatograms for standard substances protocatechuic acid and CAE, respectively.

**Figure 2:**
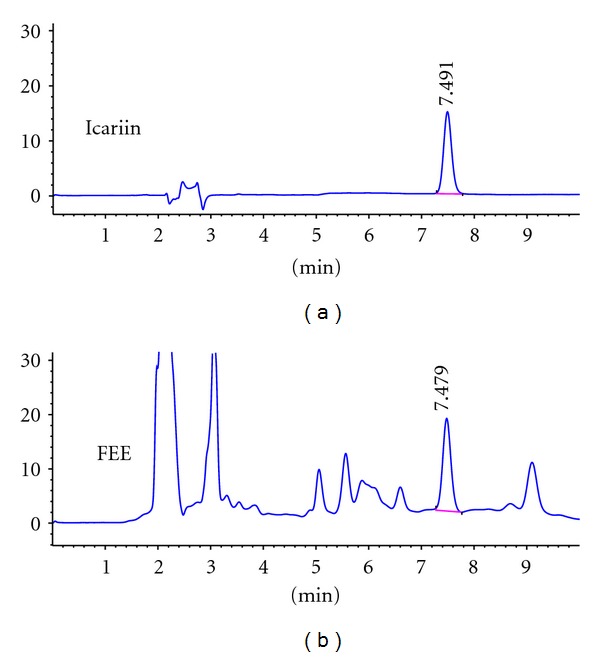
HPLC for the qualitative analysis of icariin in the *Folium Epimedii* extract (FEE). The chromatograms for standard substances and FEE are shown in the upper panel and lower panel, respectively. ((a) and (b)) The chromatograms for standard substance icariin and FEE, respectively.

Figures 1 and 2 show typical chromatographic profile including standard samples and CAE or FEE. Syringin, protocatechuic acid, and icariin were adequately resolved from other unknown compounds and could be clearly identified by retention time. The content of the three compounds in the extracts was calculated from the relevant peak areas with external standard method. The percent of syringin and protocatechuic acid in CAE and icariin in the FEE was quantified as 0.32%, 0.73%, and 0.56%, respectively.

### 2.3. Animal Grouping and Treatments

Seventy two 3-month-old virgin Wistar rats with body weight of 250 ± 20.0 gram were obtained from the Experimental Animal Center of Academy of Military Medical Sciences (Beijing, China; SCXK-(Military) 2002-001; the Institutional Ethics Committee of CACMS on animal experiment approved the experimental research on the animals). The acclimatized rats were either untouched as normal control (NC, *n* = 12) or Sham-operated (Sham, *n* = 12) or bilaterally ovariectomized (OVX, *n* = 48) using the dorsal approach [13]. The OVX rats were randomly divided into four groups: OVX group (OVX, *n* = 12); 17*β*-estradiol treatment group (E2, *n* = 12); FEE treatment group (FEE, *n* = 12); CAE treatment group (CAE, *n* = 12). The rats of E2 group received 17*β*-estradiol (Sigma, Saint Louis, MO) dissolved in small amounts of ethanol with the volume adjusted with olive oil to give a concentration of 30 *μ*g/kg body weight and were administered daily subcutaneously. The rats in FEE and CAE groups were fed with normal diet and FEE or CAE at 0.8 g/kg body weight/day (dissolved by distilled water) by oral gavage, respectively. The gavage dosages were used based on the recommended dosage for human (10 g/day) from Chinese Pharmacopeia, multiplied by the rat/human body mass ratio. 7-fold (0.2 g/kg body weight/day), 14-fold (0.4 g/kg body weight/day), and 28-fold (0.8 g/kg body weight/day) of adult dosages were used as low, medium, and high dosage of rats, respectively. In previous pilot studies (data not shown), we found that the high dosage of two aqueous extracts (0.8 g/kg body weight/day) was safe and had best effect on inhibiting bone loss. So we used high dosage of two aqueous extracts in this study. Rats in the Sham and the OVX groups were administrated with the same volume of distilled water by oral gavage. The treatment started 4 weeks after the surgery for 12 weeks. On the 15th day and the 3rd day before sacrifice, all the rats received tetracycline (Sigma, Saint Louis, MO) at 30 mg/kg body weight by intraperitoneal injection. The body weight of each rat was monitored weekly to assess effect of the treatments.

### 2.4. Preparation of Specimens

One day following the last treatment, the animals were anesthetized with intraperitoneally injected ketamine at 80 mg/kg body weight, together with xylazine at 12 mg/kg body weight, and sacrificed by exsanguination. Blood samples were obtained by abdominal aorta puncture before death and collected in heparinized tubes. Blood samples were then centrifuged at 3,000 ×g at 4°C for 10 min, aliquoted, and frozen at −80°C until used for assay. The uterus was dissected out and weighed immediately [14]. Proximal right tibiae were dissected and filled in physiological saline and stored at −20°C for measurement of bone mineral density (BMD) by dual-energy X-ray absorptiometry (DXA). After measurement of BMD, proximal right tibiae were fixed in 4% paraformaldehyde for 24 h, dehydrated in an ethanol gradient of 80%, 90% and 100% for 2 days at each step, defatted in xylene for 2 days, and embedded in plastic polymer. Undecalcified sections were stained with methylene blue or used for fluorescence morphology observation. Proximal left tibiae were fixed in 4% paraformaldehyde for 24 h and decalcified in 10% EDTA at 4°C for 3 weeks. After that, decalcified samples were dehydrated in 15% sucrose solution for 10 hours. Frozen decalcified sections were fixed in acetone and were made for immunohistochemistry and *in situ* hybridization.

### 2.5. DXA Assay

The BMD of the tibia was measured using OSTEOCORE 3 Visio by DXA (Medilink, France) according to a method previously described [15].

### 2.6. Bone Histomorphometric Analysis

Undecalcified tibial sections were used for measuring bone remodeling activity. All measurements were performed with the automated upright microscope system (Leica DMB6000B and CTR6000, Leica, Wetzlar, Germany) and image analysis system (Qwin, Leica, Wetzlar, Germany). Five bone histomorphometric indices about bone mass and bone turnover were analyzed including trabecular bone volume (BV/TV), resorption surface (ES/BS), active forming surface (MS/BS), mineral apposition rate (MAR), and osteoid seam width (O.Th). All histomorphometric indices were reported according to the standardized nomenclature recommended by the American Society of Bone and Mineral Research [16]. All animal data were obtained by blind measurements.

### 2.7. Immunohistochemical Analysis

The decalcified frozen sections were mounted on glass slides and used for immunohistochemical assessment. Protein expression of osteoprotegerin (OPG) and receptor activator of nuclear factor kappa-B ligand (RANKL) in tissue sections was detected by using anti-rat antibodies of either RANKL or OPG (Santa Cruz, Santa Cruz, CA). The sections were rinsed in TBS and immersed in 0.3% hydrogen peroxide for 5 min. The slides were then incubated with specific antibodies for 1 h at 37°C and then rinsed with TBS three times for 3 min. Sections were then incubated with the appropriated unbiotinylated secondary antibody (Zhongshan Goldenbridge, Beijing, China) for 30 min at 37°C. Slides were then treated with a solution containing DAB (1, 4-dideoxy-1, 4-imino-D-arabinitol-diaminobenzidine; Sigma, Saint Louis, MO) incubated for 3 min and rinsed by running water. After that, it was counterstained with Harris hematoxylin and then sealed. As negative control, nonimmune goat serum was used instead of the primary antibody.

All measurements were performed with the automated upright microscope system (Leica DMB6000B and CTR6000) and image analysis system (Qwin). Five random images within tibiae from two sections obtained from five rats were randomly captured with 400x magnifications. The positive immunostained area in a total area under a high-power field of each section was calculated. The positive density is percentage of positive labeled area under high-power field.

### 2.8. Preparation of Riboprobe and *In Situ* Hybridization Analysis

Total RNAs were extracted from Wistar rats bone and spleen and BALB/c mouse bone using the SV total RNA isolation system (Promega, Madison, WI, USA), and then a reverse-transcription polymerase chain reaction (RT-PCR) was performed. The primer sets were designed to amplify OPG and RANKL, respectively (Table 1). All selected regions in the mouse contain completely homologous sequences with those in the rats. The suitably digested PCR products were ligated into the pGEM-3Z Vector (Promega) to synthesize both antisense and sense probes. The ligated plasmids were then transformed into *Escherichia coli* DH5*α* competent cells, and positive clones were selected. The linearized plasmids were transcribed with T7 or SP6 polymerase and labeled with digoxigenin UTP using the DIG RNA labeling kit. All of the inserted DNA fragments were precisely confirmed by dideoxy sequencing.

The decalcified frozen sections after brief warming up in room temperature were immersed in solution of 30% hydrogen dioxide and methanol for 30 min and then incubated with pepsin diluted by 3% citric acid at 37°C. After that, the sections were postfixed in 1% paraformaldehyde for 10 min. Sections were then incubated with the DIG-labeled antisense cRNA probes at 38°C–42°C overnight in a humidified chamber. Posthybridization washes were preceded by multiple washes in 4 × SSC at room temperature. Slides were incubated in a blocking reagent for 30 min at 37°C and then incubated with a biotinylated antidigoxin antibody for 60 min, SABC for 20 min, and the biotinylated peroxydase for 20 min at 37°C in turn. Staining was performed with DAB (Sigma, Saint Louis, MO). Finally, sections were covered with glycerol gelatin and coverslips. 

All measurements were performed with the automated upright microscope system (Leica DMB6000B and CTR6000) and image analysis system (Qwin). Five random images within tibiae from two sections obtained from five rats were randomly captured with 400x magnifications. The positive immunostained area in a total area under a high-power field of each section was calculated. The positive density is percentage positive labeled area under high-power field.

### 2.9. Enzyme-Linked Immunosorbent Assay (ELISA)

Serum measurements of osteocalcin (OC), interleukin-1 beta (IL-1*β*), interleukin-6 (IL-6), calcitonin (CT), and parathyroid hormone (PTH) concentration were performed using quantitative, noncompetitive, and sandwich ELISA assay kits (Market, San Jose, CA) for detection. Absorbance was read in an ELISA reader (Bio-Tek, Colmar, France) at 490 and 540 nm as per manufacturer's instructions.

### 2.10. Statistical Analysis

All values were expressed as mean ± standard deviations (SD). All analyses were carried out using the SPSS 13.0 (SPSS Inc. Chicago, IL, USA). The difference between the groups regarding the evaluated parameters was tested by using one-way analysis of variance (ANOVA), followed by Fisher's LSD test. *P* values less than 0.05 were considered statistically significant.

## 3. Results

### 3.1. Effects of CAE on Body and Uterine Wet Weights

As shown in Table 2, the body weight of OVX group was significantly higher than Sham group. CAE remarkably inhibited the OVX-induced weight gain after treatment of 12 weeks, which is similar to E2. 

OVX caused significant atrophy of uterine tissue compared to Sham group, indicating the success of the surgical procedure. Administration of E2 significantly increased the uterine weight compared to OVX group, while FEE or CAE had no remarkable effect on uterine weight. 

### 3.2. Effects of CAE on BMD

To investigate whether CAE has an antiosteoporotic effect, the BMD of the tibia was measured by DXA.  Table 3 showed that ovariectomy significantly reduced BMD of tibia compared to Sham-operated animals. Treatment with either FEE or CAE for 12 weeks significantly increased the BMD of tibia compared to the OVX group. This effect is similar to E2.

### 3.3. Effects of CAE on Indices of Bone Histomorphometry

Bone remodeling is a life-long process where two counterbalanced processes (bone resorption and bone formation) are involved. Bone remodeling activity can be monitored by measuring histomorphometric indices. To further estimate the effects of FEE or CAE on Bone remodeling activity, we analyzed five bone histomorphometric indices including BV/TV, MS/BS, ES/BS, MAR, and O.Th in all rats using histomorphometry (see Table 3). BV/TV was significantly reduced in rats with estrogen deficiency induced by ovariectomy. Yet, MS/BS, ES/BS, MAR, and O.Th were increased significantly in these OVX rats.

E2 treatment significantly reversed the effects of ovariectomy on histomorphometric indices by increasing BV/TV and decreasing MS/BS, ES/BS, MAR, and O.Th in OVX rats. Compared to OVX rats, FEE showed a similar effect to E2 treatment on five indices, while CAE treatment only had raising BV/TV and depressing effect in ES/BS.

### 3.4. Effects of CAE on Expression of OPG and RANKL

To monitor bone remodeling activity in OVX rats treated with Chinese herbs, we analyzed expression of OPG and RANKL, two important bone remodeling factors, using immunohistochemistry and *in situ* hybridization. Further more, RANKL/OPG ratio was calculated.

**Figure 3:**
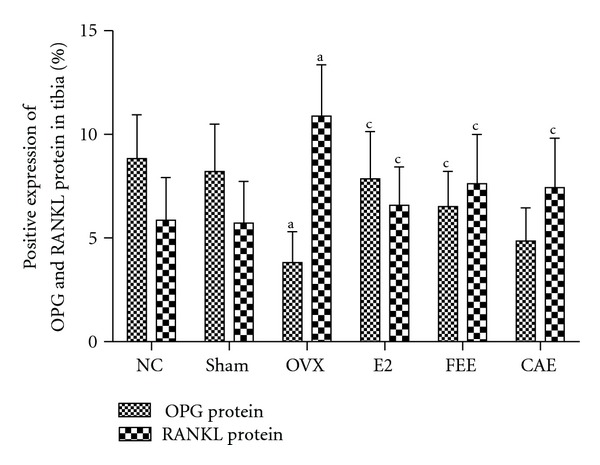
Effects of 12-week treatment with *Cortex acanthopanacis* extract (CAE) or *Folium Epimedii* extract (FEE) on protein expression of osteoprotegerin (OPG) and receptor activator of nuclear factor kappa-B ligand (RANKL) for the NC (normal control rats), Sham (rats with Sham operation), OVX (ovariectomized rats), E2 (rats treated with 17*β*-estradiol), FEE (rats treated with FEE), and CAE (rats treated with CAE) groups. Results are expressed as mean ± SD, for *n* = 12. ^a^ *P* < 0.01, against Sham group; ^c^ *P* < 0.01, against OVX group.

**Figure 4:**
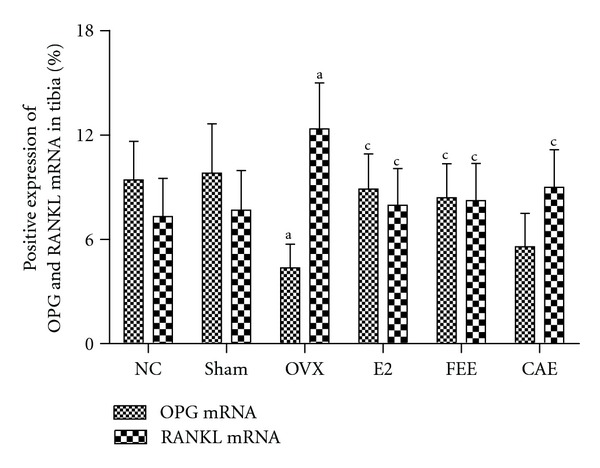
Effects of 12-week treatment with *Cortex acanthopanacis* extract (CAE) or *Folium Epimedii* extract (FEE) on mRNA expression of osteoprotegerin (OPG) and receptor activator of nuclear factor kappa-B ligand (RANKL) for the NC (normal control rats), Sham (rats with Sham operation), OVX (ovariectomized rats), E2 (rats treated with 17*β*-estradiol), FEE (rats treated with FEE), and CAE (rats treated with CAE) groups. Results are expressed as mean ± SD, for *n* = 12. ^a^ *P* < 0.01, against Sham group; ^c^ *P* < 0.01, against OVX group.

Protein and mRNA expression of OPG were decreased significantly in tibia tissue from OVX rats compared to Sham group. Either E2 or FEE treatment caused overexpression of OPG protein or mRNA in tibia from OVX rats compared to the OVX group, but treatment with CAE had no significant effects on OPG expression (Figures 3 and 4).

Ovariectomy caused a significant increase of protein and mRNA expression of RANKL in tibia compared to Sham group. Protein or mRNA expression of RANKL in tibia from OVX rats decreased significantly after treatment of E2, FEE, or CAE (Figures 3 and 4).

**Figure 5:**
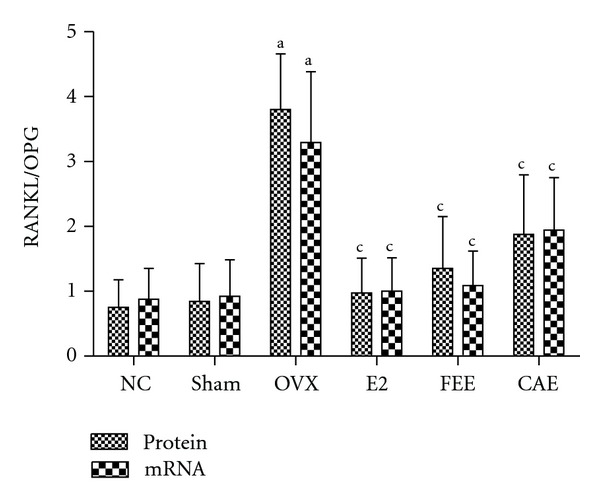
Effects of 12-week treatment with *Cortex acanthopanacis* extract (CAE) or *Folium Epimedii* extract (FEE) on ratio between receptor activator of nuclear factor kappa-B ligand (RANKL) and osteoprotegerin (OPG) expression for the NC (normal control rats), Sham (rats with Sham operation), OVX (ovariectomized rats), E2 (rats treated with 17*β*-estradiol), FEE (rats treated with FEE), and CAE (rats treated with CAE) groups. Results are expressed as mean ± SD, for *n* = 12. ^a^ *P* < 0.01, against Sham group; ^c^ *P* < 0.01, against OVX group.

RANKL/OPG expression ratio whether of protein or mRNA in tibia rose sharply in OVX group compared to Sham group, while treatment with E2, FEE, or CAE of 12 weeks could decrease significantly these ratios (Figure 5).

### 3.5. Effects of CAE on Serum Level of OC, IL-1*β*, IL-6, CT, and PTH

To determine whether CAE caused bone metabolism-related cytokines and hormones changes in serum, we examined levels of OC, IL-1*β*, IL-6, CT, and PTH in serum using ELISA (Table 4). Serum levels of OC, IL-1*β*, and IL-6 were increased significantly in OVX rats compared to Sham group, while Level of CT was decreased significantly, while E2 or FEE treatment reversed these effects substantially, and CAE treatment only caused a decrease in IL-6 and an increase in CT. Treatment with either E2 or FEE or CAE had no significant effects on PTH level in serum.

## 4. Discussion

CA and other tonic traditional Chinese herbs have been widely used for many years to treat bone diseases in China. Although these herbs have been considered as therapeutic agents to strengthen the bone by Chinese people, whether herbs have modulatory effect on bone metabolism remained unknown. In the present study, we evaluated the effect of CA on osteoporosis rats induced by OVX.

In this study, the results show that FEE or CAE treatment may significantly prevent estrogen deficiency induced the body weight gain. However, FEE or CAE did not affect the wet weight of the rats' uterus, which were atrophic and of similar weight as those of the nontreated OVX rats. The results about FEE were in agreement with other papers [8].

With an ovariectomy, BMD is markedly decreased due to an increase in bone turnover with excess resorption relative to formation in the OVX rats compared to the Sham rats, while FEE or CAE treatment increases the BMD of the tibia compared to OVX group. 

Although BMD is an important determinant of bone strength, it does not take into account bone surface remodeling activity occurring in trabecular bone. To observe the bone surface remodeling activity of cancellous bone, we had used indices of bone histomorphometry to further explain the change of BMD.

According to the results of our experiment, the OVX led to significant tibial osteopenia, as shown by BV/TV, an important bone mass index. Furthermore, coincident and significant increasing in indices for assessment of bone resorption, ES/BS, and for assessment of bone formation, MS/BS, MAR, and O.Th indicated that mature OVX rat is a good animal model for studying high-turnover osteopenia such as early postmenopausal osteoporosis. Treatment with E2 or FEE for 12 weeks was able to prevent the bone loss induced by the OVX, which was reflected by the increase in BV/TV, and lower the increased bone turnover, which was reflected by the decreases in ES/BS, MS/BS, MAR, and O.Th significantly. CAE could increase BV/TV and decrease ES/BS significantly, but the changes in MS/BS, MAR, and O.Th after treatment with CAE were insignificant compared with OVX group, which indicated that inhibitory action of CAE on bone loss lays in reducing bone resorption principally. 

The RANKL/RANK/OPG system plays a key role in the regulation of bone remodeling [17]. RANK is a receptor located on surface osteoclasts (precursor and mature). Ligands of RANK are OPG and RANKL synthesized and secreted primarily by osteoblasts and bone marrow stromal cells [18]. When RANK was activated by the RANKL, a signaling cascade is initiated, causing osteoclast differentiation and increased bone resorption. OPG, which acts as a decoy receptor for RANKL, blocks this interaction. The importance of this system in bone metabolism is demonstrated by the fact that pharmacologic blockade of RANKL is an effective treatment for osteoporosis [19], that an inherited deficiency of RANK or RANKL causes osteopetrosis, and that loss-of-function OPG mutations cause juvenile Paget's disease [20, 21]. In this study, we found that OVX could downregulate protein or mRNA expression of OPG and upregulate protein or mRNA expression of RANKL and RANKL/OPG ratio in tibia compared with Sham group, while E2 or FEE could reverse this effect induced by OVX. CAE could only significantly downregulate protein or mRNA expression of RANKL and RANKL/OPG ratio but had no significant effect on protein or mRNA expression of OPG. The results indicated that one of the mechanisms of CAE inhibiting bone loss was associated with reducing RANKL instead of raising OPG. 

Parathyroid hormone (PTH) and calcitonin (CT) are two peptide hormones that play important roles in calcium homeostasis through their actions on osteoblasts and osteoclasts, respectively [22]. *In vitro* and *in vivo* studies have clearly demonstrated that PTH increases the number and activity of osteoclasts by upregulation of RANK/RANK ligand system [23, 24]. PTH has been found to stimulate RANKL mRNA levels, to decrease OPG production, and to increase the RANKL/OPG ratio in osteoblasts/stromal cells [25]. OPG has been shown to inhibit hypercalcemia and bone resorption induced by administration of PTH [26]. Pharmacologically, CT is a hypocalcemic factor that mediates its actions through calcitonin receptor (CTR) expressed on osteoclasts [27]. CT inhibits both basal and stimulated resorption of organ-cultured intact bone, directly causes a loss of the ruffled border of osteoclasts, and reduces osteoclast number over time [28]. It has been reported that serum PTH levels were unaffected following OVX for 55 days postoperatively [29]. Our findings confirm this study since serum PTH concentrations in OVX group did not significantly differ from those seen in the Sham or treatment groups. However, OVX could attenuate level of CT in serum compared with Sham group, and E2 or FEE or CAE treatment could raise the level significantly compared with OVX group. The results suggested that inhibitory effect of CAE on bone loss was partly associated with upregulating CT.

OC, one of the very few molecules exclusively produced by osteoblasts, is a widely used marker for bone formation [30] and increases in osteoporosis of OVX rats and postmenopausal women [31]. In this study, serum levels of OC were significantly elevated in Sham animals compared to OVX group. E2 or FEE treatment had similar effect and could reduce the level significantly. However, in the group treated with CAE, serum levels of OC had no significant difference compared to OVX group, which indicated further that CAE had less effect on bone formation.

IL-1*β* and IL-6, two cytokines, were particularly important in bone resorption. IL-1*β* played a crucial role in activation of NF-*κ*B and involved in the survival of osteoclasts [32]. The importance of this signaling in normal bone physiology is demonstrated by the fact that transgenic mice deficient for IL-1*β* receptor are resistant to ovariectomy-induced bone loss [33]. IL-6, like others resorptive agents, stimulates osteoclast activity and bone resorption by an indirect mechanism, increasing interactions between osteoblasts and osteoclasts [34]. In this study, we found that OVX could upregulate the serum level of IL-1*β* and IL-6 substantially compared with Sham group, while E2 or FEE could reverse this condition significantly. However, CAE treatment could only reduce level of IL-1*β* significantly compared with OVX group rather than IL-6. The results revealed that another mechanisms of CAE inhibiting bone loss were partly associated with reducing serum level of IL-1*β*.

## 5. Conclusion

This study demonstrated that CAE had potential protective effects on OVX-induced osteoporosis in rats. CAE had inhibitory effect on resorption rather than bone formation. The preventive effect of CAE on OVX-induced bone loss depended on inhibited RANKL expression in tibia, reducing level of IL-1*β* and raising level of CT in serum. Our study provides evidence that aqueous extract of *Cortex acanthopanacis* will have potential to be used for the treatment of postmenopausal osteoporosis.

## Figures and Tables

**Table 1:** The primer sequence used in the experiments (Borster, China).

Target	Primer sequence (5′–3′)
OPG	(1) 5′-TGGAC AACCC AGGAA ACCTT TCCTC CAAAA-3′
	(2) 5′-TTTGC CTGGG ACCAA AGTGA ATGCA GAGAG-3′
	(3) 5′-AGAAA TGATA GGGAA TCAGG TTCAA TCAGT-3′

RANKL	(1) 5′-GCCAG CCGAG ACTAC GGCAA GTACC TGCGC-3′
	(2) 5′-GGCCA GGTGG TCTGC AGCAT CGCTC TGTTC-3′
	(3) 5′-TTTAT AGAAT CCTGA GACTC CATGA AAACG-3′

**Table 2:** Effects of 12-week treatment with *Cortex acanthopanacis* extract (CAE) on body weight and uterine weight.

Group	*n*	Body weight (g)	Uterine weight (mg)
NC	12	321 ± 15	834 ± 13
Sham	12	325 ± 17	832 ± 14
OVX	12	439 ± 36^a^	133 ± 8^a^
E2	12	335 ± 27^c^	820 ± 14^c^
FEE	12	371 ± 24^c^	140 ± 7
CAE	12	392 ± 28^c^	138 ± 9

Results are expressed as mean ± SD.

^a^ *P* < 0.01 against Sham group (rats with Sham operation).

^c^ *P* < 0.01 against OVX group (ovariectomized rats).

**Table 3:** Effects of 12-week treatment with *Cortex acanthopanacis* extract (CAE) on the bone mineral density (BMD), the bone volume (BV/TV), the mineralizing surface (MS/BS), the eroded surface (ES/BS), the mineral apposition rate (MAR), and the osteoid thickness (O.Th) of tibia from rats.

Group	*n*	BMD (g/cm^2^)	BV/TV (%)	ES/BS (%)	MS/BS (%)	MAR (*μ*m/d)	O.Th (*μ*m)
NC	12	0.308 ± 0.008	28.08 ± 7.26	3.56 ± 1.47	8.23 ± 2.69	1.30 ± 0.18	5.65 ± 1.34
Sham	12	0.304 ± 0.007	27.18 ± 8.78	3.40 ± 1.54	7.40 ± 2.41	1.38 ± 0.16	6.20 ± 1.29
OVX	12	0.179 ± 0.007^a^	8.95 ± 3.04^a^	9.31 ± 2.22^a^	14.54 ± 3.31^a^	1.86 ± 0.23^a^	7.77 ± 1.64^b^
E2	12	0.298 ± 0.006^c^	23.61 ± 4.71^c^	3.28 ± 1.31^c^	7.72 ± 2.66^c^	1.32 ± 0.22^c^	6.37 ± 1.42^d^
FEE	12	0.271 ± 0.007^c^	20.96 ± 4.78^c^	4.39 ± 1.38^c^	9.26 ± 2.75^c^	1.45 ± 0.19^c^	6.49 ± 1.11^d^
CAE	12	0.239 ± 0.008^d^	16.42 ± 4.05^c^	5.18 ± 1.16^c^	13.06 ± 3.70	1.74 ± 0.33	7.45 ± 1.43

Results are expressed as mean ± SD.

^a^ *P* < 0.01 against Sham group (rats with Sham operation).

^b^ *P* < 0.05 against Sham group.

^c^ *P* < 0.01 against OVX group (ovariectomized rats).

^d^ *P* < 0.05 against OVX group.

**Table 4:** Effects of 12-week treatment with *Cortex acanthopanacis* extract (CAE) on serum level of osteocalcin (OC), interleukin-1 beta (IL-1*β*), interleukin-6 (IL-6), parathyroid hormone (PTH), and calcitonin (CT) from rats.

Group	*n*	OC (ng/mL)	IL-1*β* (pg/mL)	IL-6 (pg/mL)	CT (ng/mL)	PTH (pg/mL)
NC	12	7.36 ± 1.94	243.35 ± 34.59	347.10 ± 66.50	9.88 ± 2.32	49.16 ± 12.95
Sham	12	7.06 ± 1.74	238.62 ± 40.98	361.94 ± 70.20	11.26 ± 2.80	43.53 ± 13.31
OVX	12	11.31 ± 2.41^a^	316.27 ± 35.95^a^	566.70 ± 81.54^a^	6.13 ± 1.75^a^	40.08 ± 13.14
E2	12	7.26 ± 2.02^c^	231.89 ± 44.84^c^	334.91 ± 77.28^c^	12.14 ± 3.12^c^	48.13 ± 10.94
FEE	12	8.57 ± 1.92^c^	248.59 ± 38.05^c^	400.91 ± 57.97^c^	9.26 ± 1.88^c^	46.69 ± 9.51
CAE	12	11.50 ± 2.84	279.07 ± 43.38^d^	510.10 ± 66.01	7.90 ± 2.32^d^	43.31 ± 12.80

Results are expressed as mean ± SD.

^a^ *P* < 0.01 against Sham group (rats with Sham operation).

^c^ *P* < 0.01 against OVX group (ovariectomized rats).

^d^ *P* < 0.05 against OVX group.
